# Supplementary material for: Rare Coding Variants Reveal Distinct Genetic Architectures Across Multidimensional Sleep Phenotypes
Source: medRxiv. 2026 Jun 18:2026.06.16.26355625. Preprint. [Version 1] doi: 10.64898/2026.06.16.26355625 (PMC13308334; doi:10.64898/2026.06.16.26355625)

Supplementary Information

Rare Coding Variants Reveal Distinct Genetic Architectures Across Multidimensional Sleep Phenotypes

Authors

Yingzhe Zhang[1,2,3], Wenhan Lu[4-6], Lovemore Kunorozva[3,4,7,8,], Samuel Jones[9], Matthew Maher[3,7,8], Jesse Valliere[3,7,8], Andrew R. Wood[10], Michael N. Weedon[10], Justin Tubbs[2,3,6], Konrad J. Karczewski[3-6], Tian Ge[2,3], Henning Tiemeier[11], Jacqueline Lane[3,4,7,8], Richa Saxena[3,4,7,8]*, Hanna M Ollila[3,4,7,8]*, Chia-Yen Chen[12]*[$]

1 Department of Epidemiology, Harvard T.H. Chan School of Public Health, Boston, USA

2 Center for Precision Psychiatry, Massachusetts General Hospital, Boston, USA

3 Center for Genomic Medicine, Massachusetts General Hospital, Boston, MA, USA.

4 Program in Medical and Population Genetics, Broad Institute of MIT and Harvard, Cambridge, MA, USA

5 Analytic and Translational Genetics Unit, Massachusetts General Hospital, Boston, MA, USA

6 Stanley Center for Psychiatric Research, Broad Institute of MIT and Harvard, Cambridge, MA, USA

7 Brigham and Women's Hospital Division of Sleep and Circadian Disorders, Boston, USA.

8 Department of Anesthesiology, Massachusetts General Hospital and Harvard Medical School, Boston, MA, USA.

9 Institute for Molecular Medicine Finland (FIMM), HiLIFE, University of Helsinki, Helsinki, Finland.

10 Genetics of Complex Traits, College of Medicine and Health, University of Exeter, Exeter, Devon, United Kingdom

11 Department of Social and Behavioral Sciences, Harvard T.H. Chan School of Public Health, Boston, USA

12 Biogen, Cambridge, MA 02142, USA

*These authors jointly supervised this work

[$]Current address: Merck & Co., Inc., Cambridge, MA, 02141, USA

Supplementary Figure 1. Gene discovery using gene-based (diamond), single rare variant (circle), single common variant (triangle) and meta-analyzed (square) associations for sleep phenotypes.

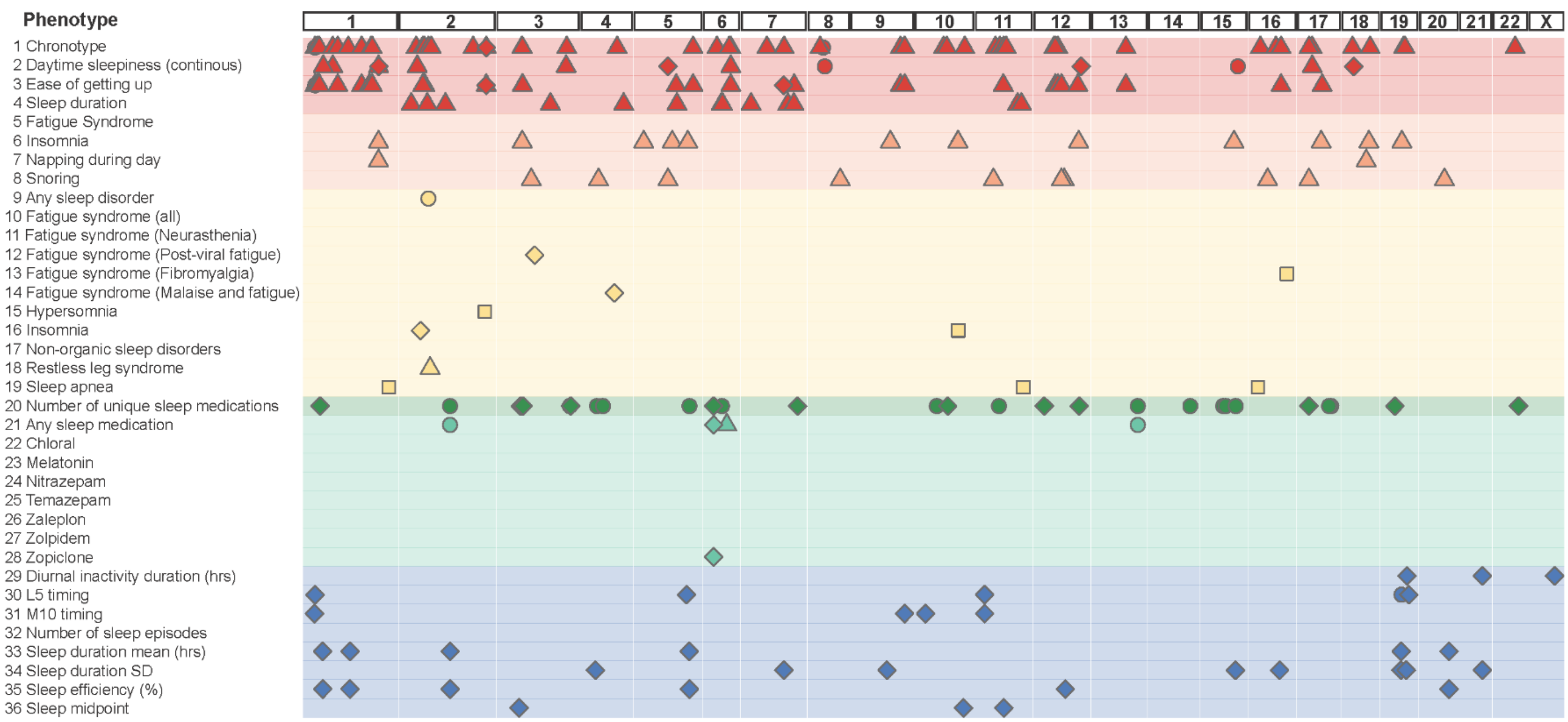

Supplementary Figure 2. Gene network enrichment in GO biological process pathways based on sleep genes. Each node represents an enriched GO term. Related GO terms are connected by a line, whose thickness reflects percent of overlapping genes. The size of the node corresponds to the number of genes, and the color of the node corresponds to the significance based on p values.

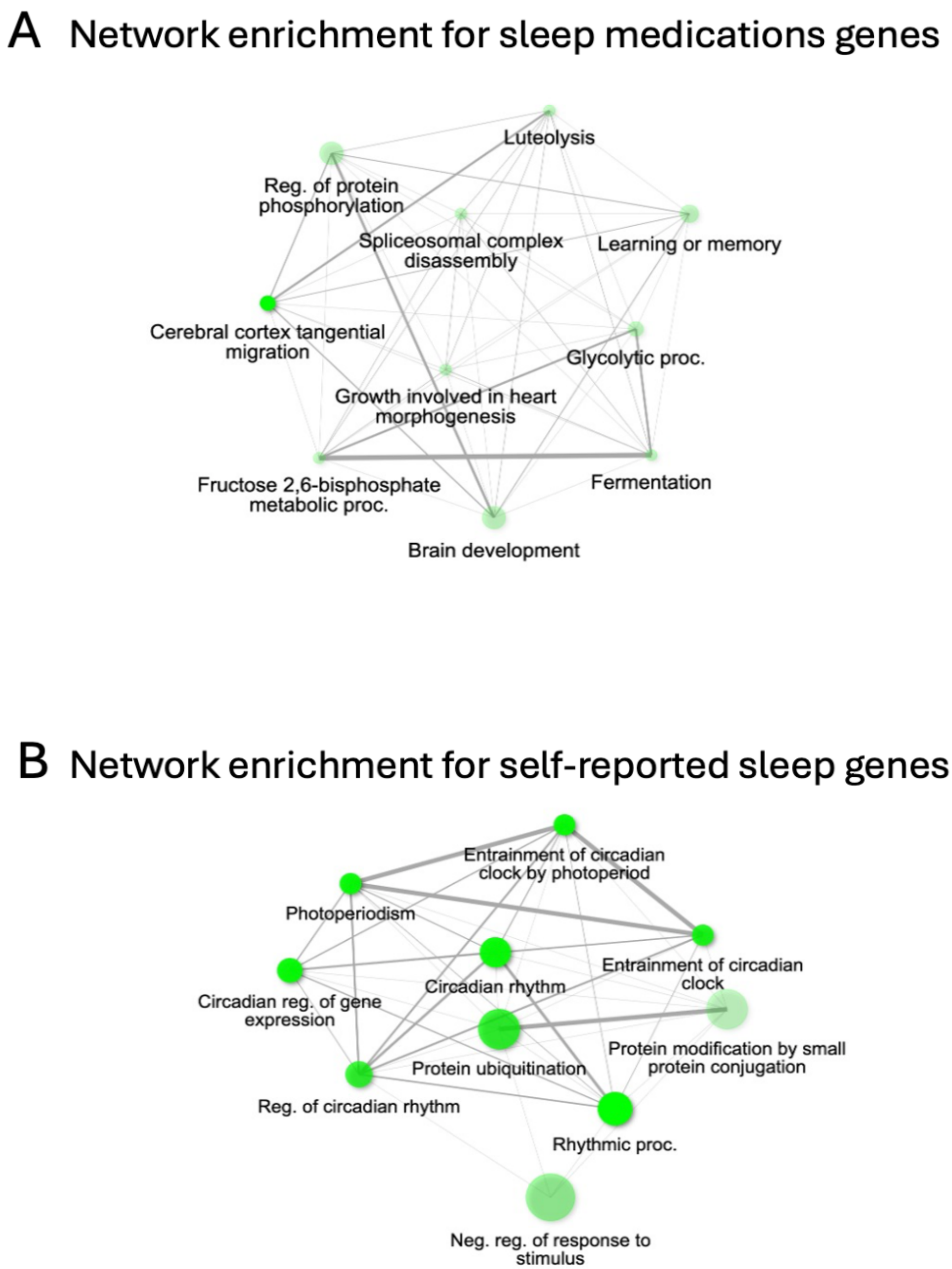

Supplement: Supplementary file 1 [file media-2.pdf]
